# A principal component analysis-based endophenotype definition for change in lung function and inhaled corticosteroid treatment response in childhood asthma

**DOI:** 10.1186/s12931-025-03426-z

**Published:** 2025-12-29

**Authors:** Shraddha Piparia, Alvin T. Kho, Brinda Desai, Richard Wong, Rinku Sharma, Juan C. Celedón, Michael J. McGeachie, Scott T. Weiss, Kelan G. Tantisira

**Affiliations:** 1 University of California San Diego, San Diego, CA USA; 2 Boston Children’s Hospital, Boston, MA USA; 3 Present address: Froedtert and MCW, Milwaukee, WI USA; 4 Present address: Phoenix Children’s Hospital, Phoenix, AZ USA; 5 Channing Division of Network Medicine, Boston, MA USA; 6 University of Pittsburgh, Pittsburgh, PA USA; 7 Rady Children’s Hospital, San Diego, CA USA

**Keywords:** Asthma, Endophenotype, Pharmacology, Corticosteroid response

## Abstract

Asthma is a clinically and biologically heterogeneous syndrome with variable symptom patterns, severity, and treatment responses. Understanding this heterogeneity is important for developing personalized management strategies. In this study, we applied principal component analysis to multiple comparable baseline clinical features in three independent pediatric asthma cohorts, CAMP (N=1,041), PACT (N=230), and GACRS (N=1,165), to define reproducible endophenotypes as quintiles of the first principal component (PC1). Across cohorts, atopy, lung function, and demographic features were the greatest contributors to variation in PC1: CAMP (67%), PACT (49%), GACRS (60%). The extremal quintiles captured consistent clinical gradients: mean pre-bronchodilator FEV1% predicted declined from Q1 to Q5, while short-acting beta agonist (SABA) usage, IgE and eosinophil levels increased. We also observed that the derived endophenotypes enhanced the ability to predict longitudinal change in lung function that is treatment-specific (inhaled corticosteroid therapy (ICS) or not) in CAMP and PACT. Together, we have (1) a PCA-based aggregation of multiple common baseline asthma clinical features for an easily implementable definition of an endophenotype that (2) stratifies pediatric asthma into clinically meaningful lung function and atopy gradients across the endophenotype group quintiles which also (3) predicts ICS treatment response - warranting a case for their use in personalizing treatment decisions.

## Introduction

Asthma is a heterogeneous syndrome with diverse clinical manifestations that can result in overlapping diagnoses and have variable responses to therapies. Its heterogeneity and subsequent disease trajectories present significant clinical challenges for long-term prognostication and treatment decision making. A sentinel unmet need is translating this heterogeneity into precision therapies tailored to individual children. Endophenotypes are distinct subtypes of a disease defined by specific biological mechanisms that allow a deeper characterization of disease heterogeneity. Despite numerous asthma endophenotype studies [1–5], several limitations hinder their clinical utility, notably the current approaches lack cross-cohort validation, or the power to predict treatment outcomes. In children specifically, Howrylak et al. [1] provided the proof of concept for data-driven clinical stratification. Moore et al. [2] and Fitzpatrick et al. [3] reported similar pediatric clusters but without cross-cohort validation or treatment prediction, whereas Spycher et al. [4] examined mixed-age populations. Carr et al. [5] focused on atypical bacterial infections rather than viral triggers most relevant to pediatric asthma.

There is a general need for practical methods for defining reproducible endophenotypes across independent study datasets with comparable clinical features. Reproducibility refers to the resulting endophenotypes having similar key clinical characteristics across nominally different study designs and populations. In addition, the ability of endotyping to anticipate longitudinal change in patient characteristics, such as lung function and symptom/exacerbation frequencies in response to treatment, will advance the personalization of care.

In this study, we apply principal component analysis (PCA) to select baseline clinical features – representing the demographics (sex, age, race), airway hyperresponsiveness, lung function, atopic status and systemic inflammation – in pediatric asthmatic populations with 3 key results: (1) the ability to define reproducible endophenotypes across independent cohorts that have (2) consistently similar key baseline clinical characteristics and association with baseline disease severity metrics (e.g., lung function and atopy prevalence), and with the (3) ability to predict response to inhaled corticosteroid (ICS) treatment as measured by longitudinal change in lung function, across multiple independent cohorts. PCA is a standard linear transformation for summarizing high dimensional (number of features) data into a lower dimensional data representation that maximizes the total data variance in this new representation [6, 7]. In contrast to previous clustering approaches [1–5] for endophenotype definition that could be sensitive to cohort-specific characteristics and model parameters, PCA components can be consistently derived in different populations, and provide a common context for cross-cohort validation. Our approach addresses the critical need for practical and generalizable endophenotype definitions that advance applications for clinical decision-making and asthma precision medicine [8].

## Methods

### Study populations

The study populations were pediatric patients diagnosed with asthma from 3 independent cohorts: Childhood Asthma Management Program (CAMP), Pediatric Asthma Controller Trial (PACT) and Genetics of Asthma in Costa Rica Study (GACRS). CAMP and PACT enrolled children with mild-to-moderate persistent asthma, whereas GACRS recruited a community-based sample regardless of severity. CAMP and PACT were interventional trials with follow-ups of 48 months and 48 weeks respectively. GACRS was an observational cross-sectional study.

CAMP [9, 10] was a randomized, placebo-controlled clinical trial of 1,041 children with mild to moderate asthma, initially aged 5 to 12 years, who were randomized to treatment with inhaled budesonide (an inhaled corticosteroid, ICS) (N=311), inhaled nedocromil (N=312), or placebo (N=418) twice daily for 48 months. We excluded the nedocromil treatment group for longitudinal analysis.

PACT [11] included 230 children with mild to moderate asthma, aged 6 to 14 years, who were randomized to treatment with fluticasone (ICS) (N= 78), fluticasone and salmeterol (LABA) (N=74), or leukotriene receptor antagonist (montelukast) (N=78) to evaluate asthma control over 48 weeks. We excluded the ICS + LABA treatment group for longitudinal analysis.

GACRS [12] was a cross-sectional study of 1,165 children aged 6 to 14 years with ancestry in the Central Valley of Costa Rica and asthma (defined as a physician’s diagonsis of asthma and either at least two respiratory symptoms or a history of asthma attack in the prior year) who were recruited from February 2001 to July 2011. GACRS was used to further assess the generalizability of the baseline endotypic features.

### Clinical features

Demographic, clinical, and atopic characteristics were collected for all study subjects at a baseline timepoint. These include sex, race/ethnicity, age at enrollment, age of asthma onset, lung function (spirometry) pre- and post-bronchodilator (pre-, post-BD), asthma exacerbation history (number of emergency room visits and hospitalization for asthma in the past 12 months), and atopy related features (presence of atopic dermatitis, hay fever, skin prick tests for common allergens and immunoglobulin E (IgE) levels). In CAMP and PACT, the % predicted pre-bronchodilator (pre-BD) forced expiratory volume in 1 second (FEV1, FEV1%pred) representing lung function, was also collected for longitudinal analysis. All three cohorts were highly T2-skewed, with median IgE above 170 IU/mL and positive skin-prick tests greater than 82% (Table 1), indicating limited representation of T2-low asthma. A summary of the baseline demographic and clinical characteristics for the study populations is provided in Table 1.

**Table 1:** Demographic and clinical characteristics of asthma cohorts

Characteristic	CAMP (N=1,041)	PACT (N=230)	GACRS (N=1,165)
Sex (Female)	420 (40%)	89 (39%)	475 (40%)
Race/ethnicity			
White	711 (69%)	129 (56%)	-
Black	138 (13%)	36 (16%)	-
Hispanic	98 (9%)	43 (19%)	1165 (100%)
Other	94 (9%)	22 (10%)	-
Age (years) [IQR]	8.9 [5.2, 13]	9.6 [6.1, 14.1]	9 [5.4, 15.2]
Exacerbation			
ER Visits (past 12 months)	-	-	969 (83%)
Hospitalizations (past 12 months)	66 (6%)	-	8 (0.1%)
Atopy			
Atopic Dermatitis (Yes)	223 (21%)	98 (43%)	543 (47%)
EOS Count [min, max]	398 [1, 5248]	5 [0.3, 25.2] (sputum %)	479 [1, 3440]
IgE Levels [min, max]	436 [2, 40738]	173 [1, 3590]	408 [1, 5000]
Positive Skin Testing (Yes)	914 (87%)	188 (82%)	962 (83%)
Hay Fever (Yes)	562 (54%)	-	372 (32%)
BMI [IQR]	17.2 [12.7, 34.3]	18.6 [13.4, 45.2]	17.2 [8.2, 41.4]
Lung function			
Baseline FEV1 (% predicted) [IQR]	94 [44, 148]	99.4 [74.98, 142.25]	98.1 [31.78, 180.46]
Baseline FVC (% predicted) [IQR]	106 [97, 112]	-	104.54 [53.14, 181.43]
Baseline BDR (%) [IQR]	8 [−17, 82]	7.67 [−16.74, 32.11]	3.98 [−35.06, 48.31]

### Endophenotype derivation

To identify reproducible clinical patterns among asthmatic children, we used principal component analysis (PCA) to summarize and simplify the relationships between correlated clinical features. PCA is a dimensionality reduction method that transforms a set of correlated variables (such as age, lung function, and atopy measures) into a smaller number of uncorrelated variables called principal components (PCs). These components are ordered so that PC1 explains the greatest amount of overall variation in the clinical data, PC2 explains the next largest independent portion, and so on. In essence, PCA allows us to capture the major axes of variation that summarize how children differ across multiple clinical characteristics in one quantitative variable.

Each subject’s clinical profile was represented by a set of 20+ quantitative clinical features (e.g., age, FEV1, IgE), where categorical features (e.g., sex, race- Caucasian vs non-Caucasian, any positive skin test) were binarily encoded (0/1). The subject profile was then standardized to have a mean of 0 and variance of 1 (a z-score transformation) across all features. PCA was applied to the set of all subject baseline clinical profiles in each cohort to identify the main directions of global clinical variation in each cohort population. The first principal component (PC1) represented the dominant axis of global clinical variation. Subjects were grouped into quintiles (five equal-sized groups, Q1–Q5) based on their continuous PC1 scores, with Q1 corresponding to the lowest and Q5 to the highest ends of this spectrum. We define Q1-Q5 to be ordinal endophenotypes that reflect gradations in global asthma clinical severity.

### Statistical analyses

We first examined how the derived endophenotypes (Q1–Q5) relate to baseline clinical features in each cohort. Associations were tested using linear regression models with Q1–Q5 membership coded as an ordinal variable (1–5). In the CAMP and PACT cohorts, we also assessed the relationship between Q1–Q5 membership and the one-year longitudinal change in pre-bronchodilator FEV1%pred, adjusted for inhaled corticosteroid (ICS) treatment versus non-ICS treatment, i.e., {longitudinal change in pre-BD FEV1%pred relative to baseline} $\sim$ 1 + {Q1 to Q5 membership} + {ICS/non-ICS treatment}.

We next compared subjects in the extremal quintiles (Q1 vs. Q5) within each treatment group to identify clinical features that differed most across the spectrum of baseline variation. Rank-sum tests were used for continuous or ordinal valued features and Fisher’s exact tests for binary features. Additionally in CAMP and PACT, we looked at differences in longitudinal FEV1%pred change between Q1 and Q5, adjusted for ICS treatment status.

## Results

### Endophenotypes defined from PC1 score quintiles have reproducibly similar key baseline clinical characteristics (magnitude and directionality) across cohorts

In each asthma cohort, we used principal component analysis (PCA) to summarize shared patterns among the baseline clinical features. This approach identifies the main axis of variation (PC1) that best captures overall differences between participants based on their clinical profiles. Each subject was then assigned a PC1 score reflecting their position along the main axis of clinical variation at baseline. We divided these scores into five equal groups (quintiles, Q1–Q5), representing ordered endophenotypes that capture gradations in overall asthma presentation, including severity-related features.

**Fig. 1:**
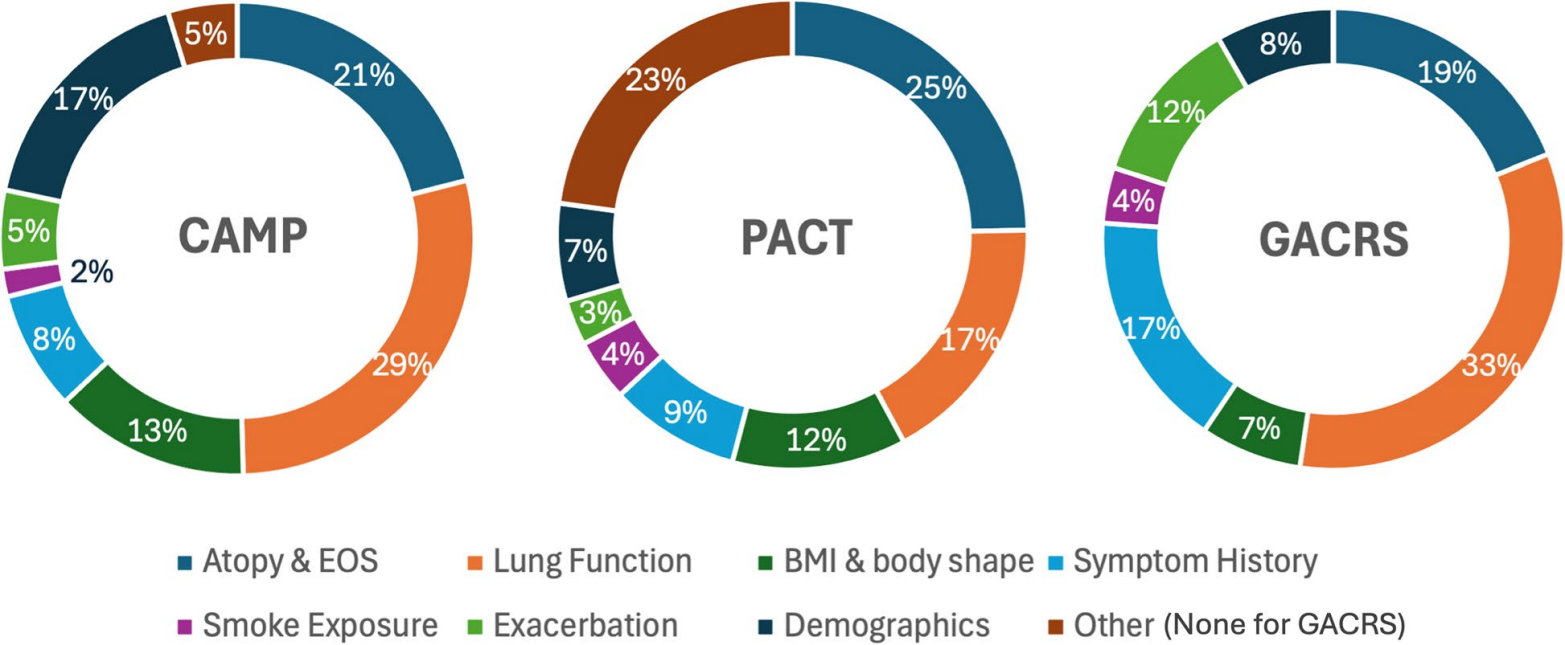
Doughnut charts showing the relative contribution of clinical feature categories to the first principal component (PC1) in CAMP, PACT, and GACRS. The figure shows category-level normalization: variable loadings were first averaged within each clinical category and then divided by the total sum of category-level means. The similar distributions across cohorts indicate reproducible patterns of variation primarily driven by demographics, lung function, and atopy. No clinical features in GACRS were categorized as “Other” in GACRS

To understand which baseline clinical features defined each endophenotype (Q1–Q5), we examined how much each feature contributed to PC1. In PCA, PC1 represents a weighted combination of all baseline features, where the loading coefficient of each feature indicates its influence on overall variation among participants. We grouped these features into four clinically interpretable categories: “demographics” (e.g., sex, race, age), “atopic status” (e.g., blood IgE level, positive allergen skin prick test, history of hay fever), “lung function” (e.g., pre-BD FEV1%pred, ratio of FEV1 over forced vital capacity (FVC)) and “recent asthma exacerbations” (emergency room (ER) or hospitalization for asthma). Figure 1 summarizes the proportionate contribution of each clinical category to the first principal component (PC1) across the three cohorts. The figure displays the category-level contributions, where feature loadings were first averaged within each category before normalization to compute percentage contribution. We matched similar clinical features across cohorts as closely as possible. Some features like allergen skin prick test were not identically characterized in all cohorts. The “Other” category consists of Inflammation markers which appeared in CAMP and PACT but not in GACRS, and was driven primarily by FeNO in PACT (loading magnitude ~0.316) and by blood lymphocyte% in CAMP (~0.072), blood neutrophil% contributed minimally (~0.022). No GACRS features were classified as “Other,” resulting in 0% for this category. Consistent with a T2-high profile, median IgE exceeded 400 IU/mL in CAMP and GACRS, and more than 82% of children were skin-test positive.

The most significant contributors to PC1 in CAMP were from 3 categories: atopic status (21%), lung function (29%), and demographics (sex, age, race) (17%), altogether representing 67% of the total of PC1 loading magnitudes. Similarly, the main clinical category contributors to PC1 in PACT and GACRS were atopic status, lung function, and demographics (sex, age, race), which represent 49% and 60% of total PC1 loading magnitudes respectively, see Fig. 1.

Moreover, subject PC1 scores (and therefore Q1-Q5 membership) were associated with key baseline clinical features in the same direction/trend from Q1 to Q5 in all 3 cohorts, Table 2. In the “demographics” category, relative to Q1, subjects in Q5 were more likely to be male (in all 3 cohorts), non-white (in CAMP and PACT), higher BMI (in CAMP and GACRS) and older (in CAMP and GACRS). In the “atopic status” category, subjects in Q5 were more likely to have higher incidences of positive skin prick tests and higher levels of blood IgE than subjects in Q1 in all 3 cohorts. In the “lung function” category, subjects had decreasing populational median of baseline pre-BD FEV1%pred from Q1 to Q5 in all 3 cohorts. In CAMP and GACRS, SABA usage indicates the % of regular usage whereas in PACT, it indicates total rescue use per day at follow-up (puffs).

**Table 2:** Demographic and clinical characteristics across quintiles (Q1 vs Q5)

Characteristic	Quintile 1 (Q1)	Quintile 5 (Q5)	Q1 vs Q5, *p*-value
AGE (yrs) median [IQR]			
CAMP	6.68 [5.96, 7.77]	11.09 [10.03, 12.08]	2.22E-56
PACT	10.25 [8.22, 11.89]	8.98 [7.3, 10.27]	0.0063
GACRS	7.96 [7.03, 9.44]	9.94 [8.55, 11.43]	5.81E-21
SEX (% Male)			
CAMP	51.44	63.94	0.0130
PACT	32.61	69.57	0.0007
GACRS	55.79	58.37	0.6399
BMI median [IQR]			
CAMP	16.31 [15.15, 17.55]	19.02 [17.06, 22.94]	6.61E-21
PACT	20.83 [17.37, 25.06]	17.45 [15.92, 19.94]	0.0002
GACRS	16.61 [15.35, 19.81]	17.94 [15.82, 21.34]	0.0012
RACE (% White)			
CAMP	81.73	58.17	2.20E-07
PACT	50	56.52	0.6763
GACRS	N/A	N/A	N/A
Positive Skin Test (% Yes)			
CAMP	59.62	99.52	1.39E-28
PACT	30.44	100	8.45E-14
GACRS	45.49	100	3.84E-49
Baseline pre-BD FEV1% predicted median [IQR]			
CAMP	106 [97, 112]	81 [74, 89]	3.27E-55
PACT	104.5 [98.23, 109.67]	92.67 [85.67, 100.8]	2.89E-05
GACRS	114.24 [101.36, 123.52]	83.08 [73.72, 89.94]	1.16E-65
Peripheral Eosinophils (per uL) median [IQR]			
CAMP	177 [86, 362]	548 [397, 870]	6.45E-35
PACT (% in sputum)	0.33 [0, 0.48]	1.01 [0.88, 1.15]	8.78E-15
GACRS	169 [99, 239]	740 [524, 1095]	3.33E-52
IgE (log10) median [IQR]			
CAMP	1.93 [1.49, 2.44]	3.07 [2.71, 3.4]	2.73E-47
PACT	1.41 [0.85, 1.81]	2.52 [2.25, 3.00]	2.68E-13
GACRS	1.94 [1.46, 2.53]	2.88 [2.61, 3.17]	1.11E-39
SABA Usage			
CAMP (% Yes)	37.50	72.60	7.33E-13
PACT (puffs/day)	0.5 [0, 0.77]	1.5 [0.71, 2.57]	5.83E-07
GACRS (% Yes)	68.67	95.71	3.41E-15

### ICS versus non-ICS treatment dependent associations of Q1-Q5 endophenotypes with longitudinal change in pre-BD FEV1%pred

**Fig. 2:**
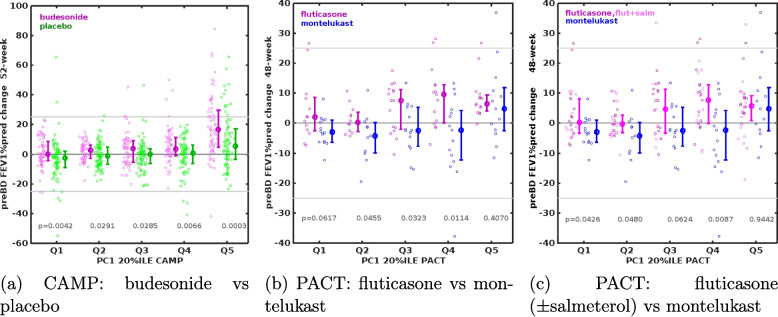
Longitudinal change in pre-BD FEV1%pred across Q1–Q5 by treatment in (**a**) CAMP: budesonide vs placebo, (**b**) PACT: fluticasone vs montelukast, and (**c**) PACT: fluticasone (± salmeterol) vs montelukast

Figure 2 shows the ~1-year longitudinal change in pre-BD FEV1% predicted across all baseline quintiles (Q1–Q5), stratified by inhaled corticosteroid (ICS) use. In CAMP, the participants receiving budesonide showed a progressive increase in lung-function gain from Q1 to Q5 (median 0.0% Q1 to 16.7% Q5), whereas the placebo (non-ICS) group showed a smaller gradient (–2.4 % to 5.5 %). The overall 52-week change in pre-BD FEV1%pred was greater in subjects receiving budesonide (beta = 4.02, $p=2.8\times 10^{-12}$) than in those on placebo (beta=2.28, $p=8.9\times 10^{-7}$). The vertical bars represent the interquartile range. Rank-sum tests comparing ICS versus non-ICS groups within each quintile supported these differences (*p*-values summarized in Fig. 2).

In PACT, the ICS group included participants treated with fluticasone or fluticasone + salmeterol (LABA), while the non-ICS comparator was montelukast. We observed that the median change in pre-BD FEV1%pred increased from 2.1% (Q1) to 6.4% (Q5) among fluticasone-treated subjects, compared with −3.0% (Q1) versus 4.7% (Q5) in the montelukast group. Consistently, ICS use was associated with greater improvement in lung function (beta=1.55, p=0.03) than non-ICS treatment (beta=1.71, p=0.06). When fluticasone and fluticasone + salmeterol were analyzed jointly as a combined ICS group, the 48-week change in pre-BD FEV1%pred remained significantly associated with Q1–Q5 membership (median 0.2 % to 5.7 %).

## Discussion

In this study, we apply principal component analysis (PCA) to select baseline clinical features - demographics (sex, age, race), airway hyperresponsiveness, lung function, atopic status and systemic inflammation – of asthmatic pediatric populations with 3 key results: (1) the ability to define reproducible endophenotypes across independent cohorts that have (2) consistently similar key baseline clinical characteristics and associations with baseline disease severity metrics (e.g., lung function and atopy prevalence), and with the (3) ability to predict ICS/non-ICS dependent treatment response specifically longitudinal change in lung function in two longitudinal trials (CAMP and PACT). Our PCA-based approach addresses limitations in current endotyping methods while providing clinically relevant insights for patient stratification and treatment selection.

The main strength of our study is the demonstration of reproducible endotyping in 3 independent cohorts, CAMP, PACT, and GACRS. Subject PC1 score quintiles were consistently used in all cohorts to define the ordinal valued endophenotypes Q1 to Q5. The main contributing baseline clinical features to these endophenotypes in CAMP were atopic status (21%), lung function (29%) and demographics (17%) altogether representing 67% of total PC1 loading. These same categories were the main contributors to total PC1 loading in PACT (49% of total) and GACRS (60% of total). Previous studies [1, 2] have identified similar features but were limited to a single cohort with no additional corroboration/validation. For instance, the SARP clustering approach identified 5 distinct clusters based on similar clinical parameters in only one population [2]. Similarly, Haldar et al. [13] identified clinical clusters but lacked cross cohort validation. Our PCA-based endophenotype definition remained consistent across heterogeneous pediatric groups, suggesting our approach to have wider application.

Our endophenotypes showed strong associations with baseline disease severity metrics across all cohorts. Specifically, subjects in Q5 consistently demonstrated features of more severe disease, including greater atopic burden and lower lung function. This aligns with previous findings by Fitzpatrick et al. [3] who identified severe asthma phenotypes characterized by similar features. Moreover, the Q5 subjects appear more likely to be male (in all 3 cohorts), non-white (in CAMP and PACT), higher BMI (in CAMP and GACRS), and older (in CAMP and GACRS). Q5 subjects also showed higher incidences of positive skin prick tests and higher levels of blood IgE compared to Q1. The relationship between lower lung function, increased SABA usage, and greater bronchodilator response remained consistent across our study cohorts. Similar associations have been reported in single-cohort studies [3], but our work uniquely demonstrates these relationships across multiple independent cohorts, supporting the robustness of our endophenotype classification.

Our endotyping also demonstrated the additional ability to predict ICS/non-ICS dependent treatment response specifically longitudinal change in lung function in CAMP and PACT. We considered 12-month budesonide change in CAMP and 48-week fluticasone change in PACT and found that the PC1 score quintile assignment predicted ICS response, with Q5 patients showing the largest bronchodilator response and greatest improvement in lung function with ICS therapy. Previous endotyping studies such as SARP and others [2, 14, 15] have included longitudinal outcomes in their endophenotype definitions but these were limited to single populations, whereas our approach is unique in its ability to predict treatment dependent responses across multiple independent cohorts. Our PCA-based endophenotype definition may serve as useful predictor of treatment response, guiding future precision medicine approaches. Since GACRS lacks longitudinal spirometry, treatment-response prediction could not be tested in that cohort.

Our current study has limitations that make it challenging to apply to any individual patient with asthma. Our major limitation is that all three cohorts were comprised of children and the result might not apply to adult populations. Previous studies have suggested that asthma phenotypes in adults are dependent on age [16]. Furthermore, the cohorts were highly enriched for T2-high inflammation as reflected in IgE levels and positive skin tests (Table 1). Thus, the results may not generalize to broader population of children with non-T2-high asthma which are prevalent in primary care. In CAMP, airway hyper responsiveness and baseline FEV1 are strongly correlated (beta=3.5, p-val $< 10^{-20}$) [17] therefore their joint contribution to PC1 is not fully independent. Also, we used retrospective data that did not have the identical baseline clinical features across the populations. We also note that the PACT cohort varied slightly in certain demographic features such as age and BMI which may be due to its smaller sample size. Some of the features used were based on the subject’s or their guardian’s self-reported responses in clinical questionnaires. In addition, the study designs of our cohorts were not identical, all of which could impact the results. However, given that our results show a general consistency across all three cohorts in terms of the key contributing baseline clinical features to the endotyping (i.e., peripheral eosinophils, lung function, atopy), we can reasonably conclude that the same approach could be applied to a general asthma population. We also recognize that our current clusters, derived from aggregate clinical score quintiles, may not fully meet the strict definition of endophenotypes. Future work will focus on validating these clinically derived clusters against molecular signatures (e.g., microRNA profiles, genetic variants such as SNPs, and other multi-omic data) to establish their biological basis and strengthen their utility as true endophenotypes.

Our results largely agree with previous clustering approaches for endophenotype definition such as by Howrylak [1] and SARP [2]. All three cohorts were recruited through clinical trials (CAMP, PACT) or specialty clinics (GACRS), creating an enrichment for T2-high asthma (greater than 82% skin-test positive with elevated IgE; Table 1). In CAMP and PACT the entry criteria (school-age children with demonstrable airway hyper-responsiveness and mild-to-moderate obstruction) further yielded predominantly male, highly atopic participants. As a result, PC1 largely re-captures these pre-selected traits and may miss subtler, T2-low patterns that are common in unselected pediatric populations. A recent population-based transcriptomic study of children aged 6–20 years reported 70% T2-low and 30% T2-high disease [18], underscoring the need to validate our framework in cohorts with a higher prevalence of T2-low asthma. Our findings on ICS responsiveness are currently limited to CAMP and PACT, and additional validation in observational cohorts with longitudinal data [19] would strengthen generalizability. Future work should apply the same PCA approach to population-based samples, incorporate viral and bacterial exacerbation data, and examine whether pathogen-response markers alter the principal components.

In summary, we have demonstrated a practical approach for reproducible endophenotype definition using PCA of select baseline clinical features with the resulting endophenotypes having consistently similar key clinical characteristics and associations with disease severity metrics, and most significantly the additional ability to predict ICS treatment response, in multiple independent asthma cohorts. This approach potentially enables more targeted treatment strategies based on endophenotype classification. Future studies should explore its application in prospective clinical trials and evaluate its applicability in guiding treatment decisions.

## Data Availability

Due to privacy concerns the dataset set can not be published publicly but interested researchers can contact the corresponding authors to collaborate on the dataset with appropriate IRB and university approvals.
